# Barriers and facilitators of physical activity for individuals with depression: a systematic review within the socio-ecological model framework

**DOI:** 10.3389/fspor.2025.1569335

**Published:** 2025-07-16

**Authors:** Marta Duina, Giampaolo Santi, Attilio Carraro

**Affiliations:** ^1^ Faculty of Education, Free University of Bozen/Bolzano, Brixen-Bressanone, Italy; ^2^ Department of Neurosciences, Biomedicine and Movement Sciences, University of Verona, Verona, Italy

**Keywords:** barriers, depression, facilitators, physical activity, socio-ecological model

## Abstract

**Introduction:**

Mental health disorders affect approximately one in every eight individuals globally, with many facing limited access to care, social exclusion, and stigma. Depression, in particular, is a leading cause of disability and has profound effects on individuals and society. Physical activity (PA) has emerged as a promising non-pharmacological intervention to alleviate depressive symptoms and promote well-being. However, people with depression often encounter substantial barriers to PA participation, exacerbating sedentary behaviors and health disparities.

**Methods:**

This systematic review synthesizes findings from 14 peer-reviewed studies (*n* = 25,375 participants examining barriers and facilitators to PA engagement among individuals with clinically diagnosed depression. Studies included both qualitative and quantitative designs and were identified through searches in PubMed, ProQuest, Web of Science, Scopus, Cochrane Library, and PsycINFO. Inclusion criteria focused on studies involving participants with depression, as defined by DSM criteria, and reporting on PA-related barriers or facilitators. Studies involving healthy populations or comorbid severe physical conditions were excluded.

**Results and Discussion:**

Adopting Bauman's socio-ecological model, there view categorizes determinants across multiple levels: individual (e.g., motivation, symptoms), interpersonal (e.g., social support), environmental (e.g., access to facilities), and policy/cultural level (e.g., educational status). Findings underscore the importance of multi-level, tailored interventions to address the complex challenges faced by individuals with depression in engaging in PA. Recommendations are provided to guide the development of inclusive, context-sensitive strategies that promote mental health through PA.

**Systematic Review Registration:**

https://www.crd.york.ac.uk/PROSPERO/view/CRD42025631996, identifier CRD42025631996.

## 1 Introduction

Mental health disorders have become one of the most significant global health challenges, affecting an estimated one in every eight individuals, according to the World Health Organization (1, 2). Depression, in particular, represents one of the most urgent public health issues worldwide, impacting an estimated 280 million people and accounting for a substantial proportion of the global disease burden, profoundly affecting individuals' quality of life, productivity, and overall well-being (2). Depression causes a pervasive low mood, reduces motivation and energy, diminishes interest in activities, and produces physical symptoms such as fatigue and sleep disturbances, all of which undermine daily functioning (3): all these impairments significantly interfere with everyday life and contribute to a marked reduction in quality of life (4, 5). Moreover, depression carries profound societal implications, not only for individuals experiencing mental health issues but also for society as a whole, affecting economic productivity, social cohesion, and the general well-being of communities (6). Therefore, identifying effective interventions to alleviate this burden represents a public health priority and requires a multi-faceted approach which integrates both pharmacological and non-pharmacological strategies. Among the latter, physical activity (PA) has emerged as a particularly promising approach for the prevention and treatment of depression (7).

A number of studies have demonstrated the positive impact of PA on depressive symptoms: it has been proved to reduce depressive symptoms, improve cognitive function, boost self-esteem, and foster social connections (8, 9). About the mechanisms underlying this effect, PA is believed to influence depression in several ways, including the modulation of neurobiological systems (e.g., increased levels of brain-derived neurotrophic factor and endorphins) and the reduction of inflammation and oxidative stress (10). Additionally, PA practice also provides psychological benefits, such as enhanced self-efficacy, distraction from negative thoughts, reduced feelings of loneliness and increased opportunities for social interaction, all of which are particularly relevant for individuals with depression (11). These benefits arise from both physiological processes and psychological factors, including the sense of accomplishment and routine associated with regular exercise (12).

However, despite this robust evidence base, adherence to PA interventions among individuals with depression remains challenging. Engaging in and maintaining regular PA presents challenges for individuals with depression, and access to PA remains unequal across different populations, with individuals suffering from mental health issues facing significant barriers to participation (13). These barriers can range from symptoms of depression itself (e.g., low energy, lack of motivation, and cognitive impairments), a lack of knowledge among health professionals and environmental, social and economic factors that further hinder PA participation (14). Stigma, discrimination, and cultural attitudes around mental health often limit social engagement in sports and access to PA, especially in certain societies. Financial constraints, inadequate infrastructure, and a lack of inclusive programs further marginalize individuals with depression, restricting their participation in activities that could enhance their well-being (14). Conversely, facilitators such as peer support, professional guidance, and community-based initiatives have shown potential in boosting participation rates (15). It is crucial for stakeholders across sectors, including healthcare, education, and sports, to collaborate in developing targeted interventions that facilitate inclusive opportunities to join physical activities for people with depression. Understanding these barriers and facilitators is essential to designing interventions that are both effective and sustainable.

Moreover, supporting PA engagement in individuals with depression may also yield indirect benefits for their immediate social environment, including family members and caregivers, who often experience emotional and logistical burdens associated with providing support (16, 17). Addressing participation challenges can thus contribute to improved outcomes not only for individuals but also for their broader support networks. To better understand these challenges and develop effective interventions, it is necessary to adopt a holistic framework that captures the interplay of individual, social, environmental, and systemic factors. For this reason, the present paper aims to delve deeper into these challenges by reviewing existing literature on the barriers and facilitators affecting PA participation among individuals with depression. To explore these barriers and facilitators in greater depth, the present review adopts the socio-ecological model proposed by Bauman (18), a framework that acknowledges the complex interplay between individual, interpersonal, environmental, and policy/cultural level factors in shaping health behaviors. It was selected for its ability to integrate multiple levels of influence, making it particularly well-suited for studying behaviors like PA, which are affected by both personal and structural conditions. Unlike more individually focused models, such as the Theory of Planned Behavior and the Health Belief Model, the socio-ecological approach encompasses a broader range of determinants (social, environmental, and policy-related) which are considered essential for understanding PA behavior. While its broad scope can pose challenges in operationalization, its comprehensiveness offers a valuable lens for synthesizing heterogeneous findings across study types and contexts (18).

By examining factors at individual, interpersonal, organizational, community, and policy/environmental levels, this review aims to offer a nuanced understanding of the multi-level influences on PA participation among individuals with depression and related mood disorders. Moreover, this review adopts the structure and purpose of the work by Vancampfort et al. (19), which also explored barriers and facilitators to PA, framed within the socio-ecological model, and highlighted the multifaceted nature of these influences, considering not only individual and interpersonal factors but also environmental and societal barriers. While the review by Vancampfort et al. provided an important foundation, it was limited to studies published up to 2015 and did not fully reflect recent developments in literature or shifts in contextual and societal factors influencing PA. In contrast, the present review aims to update and expand the existing knowledge base by incorporating more recent studies and offering a broader analysis that captures emerging barriers and facilitators for people with depression. This updated perspective is essential for informing current interventions and policy efforts that promote PA in this population.

## 2 Methods

The review protocol was registered with PROSPERO register of systematic review (CRD42025631996). This study followed a systematic review design aimed at identifying and synthesizing the barriers and facilitators to physical activity among individuals with depression. The review was conducted in accordance with the PRISMA guidelines. A comprehensive search was performed across six electronic databases using predefined keywords related to depression and physical activity. Studies were included if they involved participants with a clinical diagnosis of depression (according to DSM criteria) and reported specific barriers or facilitators to physical activity. Studies were excluded if they involved healthy populations, participants with severe physical comorbidities, or if they did not report relevant outcomes. Screening was conducted in two phases, first by title and abstract, then through full-text review. Data from the included studies were charted and analyzed qualitatively through thematic synthesis. The results were then organized using Bauman's socio-ecological model to identify and categorize key patterns across different levels of influence (individual, interpersonal, environmental, and policy/cultural).

### 2.1 Data sources

The review included a systematic search of the following electronic databases: PubMed, ProQuest, Web of Science, Scopus, The Cochrane Library, and PsycINFO. The literature search ran from 1st October 2024 to 10th January 2025, and includes publications from 2015 to present. This time frame was chosen to build upon and update the findings of previous reviews, particularly the work by Vancampfort et al. (19), which focused on earlier studies. An initial search across all databases (PubMed, ProQuest, Web of Science, Scopus, Cochrane Library, and PsycINFO) was conducted on 1st October 2024, followed by screening and evaluation of retrieved records during the following months. A second search was performed on 2nd January 2025 to capture any newly published studies, and the entire literature search process was concluded on 10th January 2025. Searching strategy included keywords such as “physical activity”, “barriers and facilitators”, “mental disorders”, and “depression”. Filters such as language (English) and time period (from 2015 to present) were applied to refine the search results. The complete research strategy, including the various search strings used for each database, can be found in the Supplementary Materials.

To complement the database search, the reference lists of some relevant systematic reviews were manually screened to identify potentially eligible studies that might not have been captured through keyword-based searches. Any articles already retrieved from the databases were excluded to avoid duplication. Although grey literature sources were consulted, no additional eligible studies were identified through those channels. After the removal of duplicates, two independent reviewers screened the titles and abstracts, followed by full-text evaluations to determine final eligibility.

### 2.2 Inclusion and exclusion criteria

The review included studies exploring barriers, facilitators, or influential factors related to PA engagement in people with diagnosed depression (e.g., major depressive disorder, dysthymia, depressive episodes) of any age, gender, or cultural background. Both quantitative (e.g., surveys, intervention trials) and qualitative studies (e.g., interviews, focus groups) were considered. Only studies involving people with depression or mood-related disorders as defined by DSM criteria were included, with a focus on studies where more than 50% of the participants were diagnosed with depression. Exclusion criteria comprised studies focusing solely on PA in populations without a diagnosis of depression, non-peer-reviewed literature that does not meet quality appraisal standards, studies involving participants diagnosed with severe metabolic, neurological, or musculoskeletal disorders, and studies that included healthy subjects or did not specifically focus on depression or mood-related disorders. To improve clarity and transparency, the inclusion and exclusion criteria are summarized in Table 1.

**Table 1:** Inclusion and exclusion criteria (structured by PICo and study type).

Element	Inclusion criteria	Exclusion criteria
Population	Individuals of any age, gender, or cultural background diagnosed with depression (e.g., MDD, dysthymia), with >50% of the sample meeting DSM criteria	Studies on individuals without a formal diagnosis of depression; samples with <50% of participants diagnosed
Interest	Studies exploring barriers, facilitators, or influential factors related to PA engagement	Studies not addressing PA-related barriers/facilitators or focusing only on general health behaviors
Context	Any setting (clinical, community, etc.)	Studies involving participants with severe comorbid conditions (e.g., neurological, metabolic, or musculoskeletal disorders)
Study Type	Peer-reviewed quantitative (e.g., surveys, intervention trials) and qualitative (e.g., interviews, focus groups) studies	Non-peer-reviewed sources, editorials, conference abstracts, or grey literature not meeting quality standards

### 2.3 Data collection

**Figure 1:**
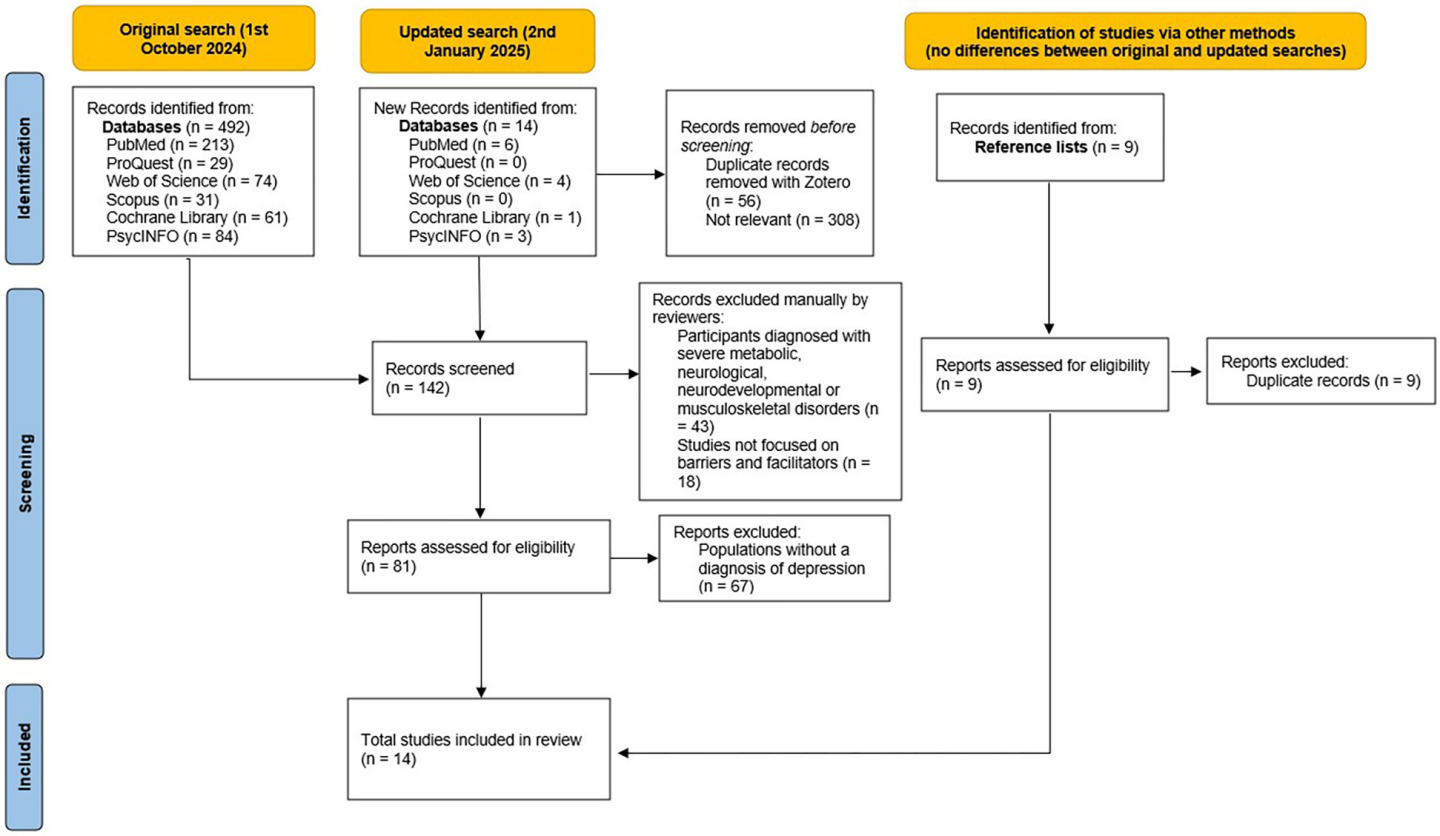
PRISMA flow diagram.

All references retrieved from the database search were imported into Zotero (version 6.0.36), which was used to manage citations and facilitate the removal of duplicates. The screening of titles, abstracts, and full texts was conducted manually and independently by two reviewers, following predefined inclusion and exclusion criteria. Reviewers were blinded to each other's decisions, and discrepancies were resolved through discussion and consensus with the third author. A PRISMA flow diagram was used to document the screening and selection process (Figure 1). Data extraction included information on study design, participant demographics, PA interventions, reported barriers and facilitators, and outcome measures. Two reviewers independently extracted data, with the third reviewer verifying the data. Disagreements were resolved through discussion with the third author. Screening decisions and data extraction were tracked using a shared Excel spreadsheet to ensure consistency and traceability throughout the review process.

### 2.4 Selection and categorization of variables

Data were synthesized in relation to the study characteristics, the strategies used in the analyses, and the outcomes.

Given the qualitative nature of the synthesis, the review team adopted a primarily descriptive approach and did not impose additional interpretation beyond what was reported in the original studies. Factors were extracted and categorized as barriers or facilitators based on how they were explicitly described by the study authors. The reviewers’ role was limited to mapping these elements within the socio-ecological framework in a structured and consistent manner.

A content analysis was conducted by dividing the barriers and facilitators to PA participation into categories, which were then analyzed according to the socio-ecological model. The identified categories were: (a) individual, (b) interpersonal, (c) physical and social environment, and (d) political/cultural factors. The socio-ecological approach aims to identify the domains explored in the literature and clarify the multidimensional perspective of potential influences on PA behavior in patients with depression (18, 19).

Nevertheless, the research team remained aware of the potential influence of their disciplinary backgrounds during data synthesis. To enhance reflexivity, key decisions were made collaboratively, and the categorization process was reviewed jointly to ensure coherence and reduce subjective bias. When discrepancies emerged during the classification of factors within the socio-ecological model, these ambiguities were discussed until consensus was reached. Such cases were considered to reflect the overlapping and interdependent nature of the model's levels, rather than inconsistencies in the data themselves.

## 3 Results

A total of 14 studies, for a total of 25,375 participants, were included in the review. The reasons for exclusion are listed in Figure 1. Of the 14 studies, 5 exclusively involved participants with depression and related mood disorders (20–24), 6 included participants in which the majority were diagnosed with depression (25–30), 1 focused on clinical professionals working with individuals with depression (31), and 2 were reviews that exclusively included patients with depression (32, 33). The main information from the selected articles is summarized in Table 2.

**Table 2:** Characteristics of included studies.

First author/year	Total participants	Age	Diagnosis	Study approach	Method
Fraser, 2015; 2016^a^	101 (*F* = 73)	*M* = 40.7 years old	Depression 61.4%	Quantitative	Self-report survey/questionnaire, Accelerometer data
Pengpid, 2015	17,928	*M* = 20.8 years old	2,178 Depression	Quantitative	Self-report questionnaire
Pentecost, 2015	15 participants and 9 clinicians	>18 years old	Depression	Mixed (Qualitative)	Interviews
Vancampfort, 2015	165 (*F* = 105)	*M* = 45.6 years old	96 Depression 69 Bipolar disorder	Quantitative	Self-report survey/questionnaire
Busch, 2016	102 (*F* = 51)	*M* = 39.4 years old	Depression	Quantitative	Self-report survey/questionnaire
Glowacki, 2017	957 participants, 13 studies	18–65 years old	Depression	Scoping review	Different study designs
Pelletier, 2017	2,678	>18 years old	1,134 with mood disorders, 576 with anxiety disorders, 968 with concurrent mood and anxiety disorders	Quantitative	Survey
Sims-Gould, 2017	24 (*F* = 16)	*M* = 52 years old	18 Major Depressive disorders 6 Bipolar disorder	Qualitative	Interviews
Radovic, 2018	125 (clinicians) (*F* = 100)	18–65 years old	Depression	Mixed	Online survey
Sunesson, 2021	14 (*F* = 10)	*M* = 17 years old	Depression	Qualitative	Interviews
Griffiths, 2022	19 (*F* = 15)	*M* = 45.12 years old	Depression	Qualitative	Interviews
Rowland, 2023	7 (*F* = 7)	55–70 years old	Depression	Qualitative	Focus group
Dai, 2024	3,238 participants, 31 studies	>60 years old	Depression	Meta-Analysis	Randomized controlled trials

^a^ Both studies are based on the same sample.

In terms of methodology, quantitative approaches were the most commonly used, with self-report surveys and questionnaires being predominant in the studies by Busch (2016), Dai (2014), Fraser (2015, 2016), Pelletier (2017), Pengpid (2015), Vancampfort (2015). However, qualitative methodologies were also frequently applied, particularly in the studies by Griffiths (2022), Pentecost (2015), Rowland (2023), Sims-Gould (2017), Sunesson (2021), which relied on interviews and focus groups to gather detailed personal insights of individuals with depression.

Regarding age demographics, the studies included a wide range of participants. For example, Pengpid (2015) focused on younger participants with an average age of 20.8 years, while Griffiths (2022) and Rowland (2023) focused on middle-aged to older adults, with average ages of 45.12 and between 55 and 70 years, respectively. Notably, Dai's (2024) meta-analysis, which incorporated 3,238 participants from 31 studies, specifically focused on older adults aged over 60, further emphasizing the diverse age representation within the research.

### 3.1 Barriers within the socio-ecological model

Table 3 highlights different barriers to PA, categorized into individual, interpersonal, environmental, and political/cultural factors. At the individual level, challenges include above all depressive symptoms (such as low motivation and fatigue) cited by the majority of studies (Fraser (2015), Pengpid (2015), Pentecost (2015), Vancampfort (2015), Busch (2016), Glowacki (2017), Sunesson (2021), Griffiths (2022), Rowland (2023)), following by “Lack of drive/Lack of will power/self-discipline/Intentions” and “Physical health issues, disabilities”. “Lack of self-esteem”, “Mental illness” and “Age” also turned out to be relevant.

**Table 3:** Barriers within the socio-ecological model.

Level	Barriers	Studies
Individual	Depression symptoms (Lack of motivation, Low mood, Fatigue, Social anxiety, Exhaustion, Overwhelming tiredness, Lack of energy)/Internal emotions/Feeling states/Negative affect	(20–25, 28, 30, 33)
	Lack of confidence in the ability to exercise/Lack of self-esteem/Negative view of the self/Beliefs about capabilities	(20, 21)
	Do not find exercise enjoyable/Find exercise boring	(20)
	Sedentary behaviour	(26)
	Preference for other therapies	(22)
	Inability to build a routine	(29)
	Lack of drive/Lack of will power/self-discipline/Intentions	(24, 27, 33)
	Mental illness	(23, 25)
	Physical health issues, disabilities	(21, 25, 27)
	Motivational obstacles	(21)
	Higher BMI	(30)
	Age	(28, 30)
	Medical side effects	(25)
	Lack of knowledge about exercise prescription/recommendations	(31)
Interpersonal	Lack of encouragement and support from family/friends	(20, 24)
	Fear of intrusion and being undermined by others	(21)
	Limited time with health professional	(25)
	Exercise prescription delivered by exercise professionals	(31)
Physical and social environment	Logistical difficulties in services/Restricted access to resources	(22, 25, 30, 33)
	High cost	(27, 29)
	Lack of structural support	(24, 25)
	Environmental/external influences/context	(21, 23, 33)
	The season and weather	(21)
	Not want to go out or leave the house	(21)
Political/cultural factors	Do not have enough time/Time constraints/Too busy	(20, 21, 27)
	Complexity of materials provided	(22)
	Lower educational status	(30)
	Belief that adolescents will not adhere to an exercise	(31)

At the interpersonal level, a lack of support from family and friends is the most mentioned [in Busch (2016) and Sunesson (2021)].

In the environmental and social context, logistical difficulties and limited access to resources are covered in Fraser (2015), Pentecost (2015), Vancampfort (2015), Glowacki (2017), but also “Environmental influences/context”, “Lack of structural support” and “High cost” seem to be strongly considered. Seasonal and weather influences are discussed in Griffiths (2022), who also mentions the desire to stay home as a barrier. Finally, at the political and cultural level, the “Lack of time” is discussed in Busch (2016), Griffiths (2022), and Pelletier (2017).

### 3.2 Facilitators within the socio-ecological model

Table 4 identifies various facilitators that promote engagement in PA, categorized according to the socio-ecological theory factors. At the individual level, key facilitators include “Acknowledgement of the positive impact of exercise/Belief about consequences” as mentioned by Griffiths (2022), Fraser (2015), Glowacki (2017), and “Motivation and positive beliefs”, highlighted by Rowland (2023), Vancampfort (2015).

**Table 4:** Facilitators within the socio-ecological model.

Level	Facilitators	Studies
Individual	Perception of the benefits of physical activity (improved mood and routine)	(22)
	Building a routine in daily life	(29)
	Greater self-esteem	(24)
	Motivation and positive beliefs	(23, 30)
	Personal physical mobility	(23)
	Acknowledgement of the positive impact of exercise/Belief about consequences	(21, 25, 33)
	Has to/Need	(21)
	Positive affect	(30)
	Autonomy	(30)
	Competence	(30)
	Controlled weight	(25)
	Maintain good health	(25)
	Manage stress and improve emotional wellbeing	(25)
	Feeling states/emotions	(33)
	Behavioural regulation	(33)
Interpersonal	Support from health professionals/family/friends	(21, 23, 26, 30)
	Being socially connected with family and friends/Social influences	(25, 29, 30, 32, 33)
	Companionship while exercising	(24, 25, 32)
	Advice from a doctor or other health professional/Prescription	(27, 31)
	Exercise with personal trainers and exercise physiologists	(25)
Physical and social environment	Structured routines in inpatient settings	(25, 26)
	Accessibility of walking as a low-barrier activity, well-suited with capabilities and interests	(23, 25, 26, 32)
	Self-monitoring tools such as pedometers and motivational diaries	(22)
	Exposure to nature	(29)
	Supportive environment	(24)
	Exercise coaching	(24)
	Clinicians agreed with the benefits of exercise	(31)
	Combining group and individual activities based on individual needs and preferences	(32)
	Restructuring the social environment	(33)
Political/cultural factors	None identified	None identified

At the interpersonal level, “Support from health professionals/family/friends” plays a significant role, as indicated by Fraser (2016), Rowland (2023), Griffiths (2022), Vancampfort (2015). Additionally, “Being socially connected with family and friends/Social influences”, as discussed by Sims-Gould (2017), Vancampfort (2015), Fraser (2015), Dai (2024), Glowacki (2017), are recognized as key facilitators. The advice and prescriptions from health professionals, particularly doctors, are also highlighted as important factors (Pelletier, 2017; Radovic, 2018).

Regarding the environment, “Accessibility to low-barrier activity, well-suited with capabilities and interests” are identified as crucial facilitators (Fraser (2016), Rowland (2023), Fraser (2015), Dai (2024)). “Structured routines in inpatient settings” further encourage PA, as seen in studies by Fraser (2015, 2016). Lastly, while political/cultural factors were not specifically identified in the studies, the other facilitators emphasize the importance of a supportive and structured environment for promoting PA.

## 4 Discussion

Findings from this review highlight the complexity of the factors that influence PA participation among individuals with depression. The socio-ecological model (18), which systematize these factors into individual, interpersonal, environmental, and political/cultural dimensions, provides a comprehensive framework for understanding the diverse barriers and facilitators that shape participation in PA. This approach shows the importance of addressing PA engagement holistically, recognizing that interventions must be tailored to overcome a plethora of obstacles at multiple levels.

At the individual level, several barriers stand out. Depressive symptoms, such as low motivation, fatigue, and negative affects, create significant challenges to engaging in PA, which is consistent with the findings of Firth et al. (14), who highlighted similar barriers across various mental health disorders. Moreover, a lack of self-esteem, negative beliefs about one's abilities, and difficulties in establishing regular routines further reduce participation rates. These factors, compounded by physical health issues and mental illness (21, 23, 25, 27), highlight the need for personalized interventions which consider these emotional and cognitive challenges. However, positive individual factors, such as greater self-esteem, motivation, and belief in the positive impact of exercise (23, 30), can help people with depression take part in PA. Building self-efficacy and fostering a sense of accomplishment through regular PA may be effective strategies for improving adherence and motivation.

At the interpersonal level, support from family, friends, and health professionals emerges as a key facilitator of PA (21, 23, 26, 30). Social influences and companionship during exercise (24, 25, 29, 30, 32, 33) can help fight social isolation and provide the encouragement needed to engage in PA (20, 24). The influence of health professionals, particularly in the form of exercise prescriptions and guidance (27, 31), is also critical. However, interpersonal barriers such as fear of intrusion or being undermined by others (21) may hinder participation. Addressing these interpersonal dynamics is important to create supportive environments where individuals feel comfortable engaging in PA without fear of judgment.

Environmental factors also play a significant role in shaping participation. Accessibility to PA opportunities and the presence of supportive environments are key facilitators (23, 25, 26, 32). Programs designed to accommodate individual capabilities and preferences, such as self-monitoring tools and accessible low-barrier activities, can help individuals with depression overcome logistical barriers and participate more consistently. Moreover, the structured routines in inpatient settings (25, 26) and exposure to nature (29) have been shown to promote engagement in PA. However, logistical difficulties, lack of resources, and high costs remain persistent barriers that need to be addressed through community-based initiatives and inclusive programming.

Finally, political and cultural factors can significantly impact participation. Although the literature did not identify any specific facilitators related to these factors, several barriers were found. The most common is time constraints, with many individuals believing they do not have enough time to engage in PA due to their busy schedules (20, 21, 27). Lack of time is a common barrier also among all other individuals (34, 35). This issue is particularly prevalent in cultures where people are often overwhelmed by work, family, or other responsibilities, making it difficult to prioritize exercise.

Confronting the results of this review with Vancampfort et al. (19), several similarities emerge, particularly regarding the barriers to PA. Both reviews identify elevated depressive symptoms, higher body mass index (BMI) and physical comorbidity as key factors. Additionally, emotional and cognitive challenges, such as low self-esteem and fatigue, are recognized as critical factors limiting engagement in PA. Regarding facilitators, both reviews stress the importance of tailoring PA programs to individual limitations, echoing the need for accessible and adaptive interventions. Vancampfort et al. (19) also emphasize the role of better physical performance and higher quality of life in motivating participation, noting the importance of self-enhancement and self-determination strategies, which align with the need for self-efficacy and accomplishment discussed in this review.

Moreover, while this review primarily aimed to map barriers and facilitators to PA among individuals with depression, it also reveals population-specific trends, suggesting that certain challenges and supports may differ across age groups. For instance, in studies focusing on adolescents and young adults (24, 28), individual-level factors such as social anxiety, low motivation, and lack of drive were particularly prominent. These findings align with Radovic's study (31), which reflects clinicians' perceptions that adolescents with depression are often seen as unwilling to participate in PA programs, highlighting a potential match between perceived and actual barriers in this group. In contrast, studies involving older adults (23, 32) emphasized the critical role of social support and the importance of tailoring activities to personal interests and functional capacities. These observations suggest that while some barriers are common across groups, effective interventions may benefit from age-specific adaptations to better address the distinct needs, motivations, and challenges of each subgroup.

Building on these findings, this review offers important considerations for the design and implementation of PA interventions targeting individuals with depression. A multi-level approach, informed by the socio-ecological model, can guide the development of more inclusive, flexible, and sustainable programs. At the individual level, strategies such as motivational interviewing, goal-setting, and self-monitoring tools may help enhance self-efficacy and overcome emotional and cognitive barriers. At the interpersonal level, involving peers, caregivers, or exercise mentors can foster a sense of belonging and reduce social isolation. Healthcare professionals play a key role and should be trained to deliver tailored exercise advice and support adherence through consistent follow-up. At the environmental level, programs should prioritize accessibility by offering free or low-cost options, transportation support, and flexible scheduling. Designing safe, inclusive, and culturally appropriate physical environments (both indoor and outdoor) can further encourage engagement. Finally, at the policy and cultural level, efforts should focus on integrating PA promotion within mental health services, increasing funding for inclusive sport programs, and launching public awareness campaigns to reduce stigma around mental illness and exercise.

Operationalizing these barriers and facilitators means embedding them into each stage of intervention development, through needs assessments, user-centered design, and participatory approaches involving people with lived experience. In doing so, stakeholders can ensure that interventions are not only theoretically sound, but also practically relevant and context-sensitive.

## 5 Limitations

This review provides valuable insights into the barriers and facilitators of PA participation among individuals with depression, though several limitations should be considered. Most studies are from high-income countries, limiting the applicability of findings to low- and middle-income contexts with different cultural attitudes and systemic challenges. Additionally, many studies rely on self-reported measures of PA and depressive symptoms, which can introduce biases that can affect the reliability of results.

The heterogeneity of the studies, including variation in intervention types, outcome measures, and participant characteristics, makes it difficult to draw widely applicable conclusions. Moreover, while the socio-ecological model is a comprehensive framework, it may not fully capture the dynamic interactions between individual, social, and environmental factors in diverse settings.

## 6 Conclusion

Our findings emphasize the need for a multi-level approach to increasing PA participation among individuals with depression. Interventions must consider the complex network among individual, interpersonal, environmental, and political/cultural factors that impact engagement. This review highlights the importance of addressing both the barriers to and facilitators of PA, as well as the necessity of a holistic approach that includes targeted strategies at each level of the socio-ecological model. Developing collaborative interventions that integrate support from health professionals, communities, and policymakers, while also providing accessible and inclusive opportunities, will be crucial to improving the well-being and quality of life for individuals with depression. Future research should continue to explore these multi-dimensional influences and refine interventions based on the experiences and needs of those living with depression.

## Data Availability

The raw data supporting the conclusions of this article will be made available by the authors, without undue reservation.
