# Supplementary material for: Barriers and facilitators of physical activity for individuals with depression: a systematic review within the socio-ecological model framework
Source: Front Sports Act Living. 2025 Jul 16;7:1569335. doi: 10.3389/fspor.2025.1569335 (PMC12311504; doi:10.3389/fspor.2025.1569335)
Supplement: Supplementary file 1 [file Table1.docx]

**Research Strategy**

| **Database** | **Search query** | **Articles found** |
| --- | --- | --- |
| **Pubmed** | “barriers and facilitators AND physical activity AND mental disorders OR depression” | **219** |
| **ProQuest** | “Barriers and facilitators AND physical activity AND mental disorders OR depression” in ABSTRACT | **29** |
| **WOS** | “Barriers and facilitators AND physical activity AND mental disorders OR depression” in TOPIC | **78** |
| **Scopus** | “Barriers and facilitators AND physical activity AND mental disorders OR depression” in TITLE-ABSTRACT | **31** |
| **Cochrane Library** | “Barriers and facilitators AND physical activity AND mental disorders OR depression” in TITLE-ABS-KEY | **62** |
| **PsycInfo** | “Barriers and facilitators AND physical activity AND mental disorders OR depression” | **87** |

***Timeframe: 2015 to present**

Reference lists checked:

- Vancampfort D, Stubbs B, Sienaert P, Wyckaert S, Hert MD, Rosenbaum S, et al. What are the factors that influence physical activity participation in individuals with depression? A review of physical activity correlates from 59 studies. Psychiatr Danub. 2015;27(3):210–24. (**2 articles found**)
- Firth J, Rosenbaum S, Stubbs B, Gorczynski P, Yung AR, Vancampfort D. Motivating factors and barriers towards exercise in severe mental illness: a systematic review and meta-analysis. Psychol Med. 2016 Oct;46(14):2869–81. (**4 articles found**)
- Glowacki K, Duncan MJ, Gainforth H, Faulkner G. Barriers and facilitators to physical activity and exercise among adults with depression: A scoping review. Ment Health Phys Act. 2017 Oct;13:108–19. (**3 articles found**)
